# Detrusor underactivity versus bladder outlet obstruction clinical and urodynamic factors

**DOI:** 10.1590/S1677-5538.IBJU.2019.0402

**Published:** 2020-02-20

**Authors:** Jefferson Kalil, Carlos Arturo Levi D'Ancona

**Affiliations:** 1 Universidade Estadual de Campinas Escola de Ciências Médicas CampinasSP Brasil Escola de Ciências Médicas, Universidade Estadual de Campinas - Unicamp, Campinas, SP, Brasil

**Keywords:** Urodynamics, Urinary Bladder, Underactive, Lower Urinary Tract Symptoms

## Abstract

**Objectives:**

To evaluate the lower urinary tract symptoms, classified by the International Prostate Symptom Score (IPSS), urodynamic results (Watts Factor (WF), Bladder Contractility Index (BCI), and post void residual (PVR), in order to differentiate Detrusor Underactivity (DU) from Bladder Outlet Obstruction (BOO).

**Methods:**

Retrospective observational study performed from 2011 to 2018 at the Hospital das Clínicas of Unicamp. Two phases were done: first, to estimate sample size, and second, to evaluate the predicted parameters. Male patients with range age from 40 to 80 years were included. Patients were divided into two groups: Group 1, without BOO and with DU; Group 2, with BOO. Variables analyzed: age, comorbidities, symptoms, urodynamic data (BCI and WF) and PVR.

**Results:**

Twenty-two patients were included in each group, with medians of 68 (Group 1) and 67.5 years old (Group 2) (p = 0.8416). There was no difference for comorbidities. In relation to IPSS, medians were: 16.5 and 20.5, respectively (p = 0.858). As for symptoms, there was predominance of combination of storage and voiding symptoms in the two groups (p = 0.1810). Regarding PVR, 15 patients in Group 1 and 16 in Group 2 presented PVR> 30mL (p = 0.7411). BCI presented median values of 75 and 755.50 for Group 1 and Group 2, respectively (p <0.0001), while WF had medians of 22.42 and 73.85 (p <0.0001).

**Conclusion:**

Isolated symptoms, classified by IPSS and PVR, could not differentiate patients with DU from those with BOO, but it was possible using urodynamic data.

## INTRODUCTION

Detrusor underactivity (DU) according to the International Continence Society (ICS) includes low detrusor pressure or short detrusor contraction time, usually in combination with a low urine flow rate resulting in prolonged bladder emptying and/or failure to achieve complete bladder emptying within a normal time span (1). This definition, although adequate, does not show the parameters that define this diagnosis: method used to measure strength, value of normality and appropriate urination time.

In general, the most prevalent symptoms in men with DU are: reduction and/or interruption of urinary flow, hesitancy, incomplete emptying sensation, palpable bladder, absence and/or reduction of sensitivity, and effort to urinate (2). Recently, similarly to what occurs with the Overactive Bladder, a new clinical syndrome has been suggested, called Underactive Bladder Syndrome, which presents the following symptoms: prolonged urination time with or without feeling of incomplete emptying, usually with hesitancy, reduced sensation of filling, and weak stream (3). Nevertheless, neither the symptoms nor the urodynamic criteria that define DU are well defined so far. This is important, since the prevalence of DU is 9% to 23% in men and 12% to 45% in women (4). Differentiating patients with DU associated with bladder outlet obstruction (BOO) from those who present only bladder outlet obstruction is relevant, since the former group may not benefit from surgical treatment, while the latter will certainly do (5). Moreover, patients with DU and chronic urinary retention may develop overflow incontinence and reduced urination output, which are mild complications, in addition to severe complications, such as urinary lithiasis, recurrent urinary tract infections and renal insufficiency (6).

Regarding the diagnosis, it is necessary to use urodynamics, which is an invasive investigation. Classically, detrusor strength has been evaluated by means of pressure/flow studies. It is noteworthy that current methods used to evaluate the detrusor function are: Schafer nomogram (pressure/flow), Bladder Contractility Index (BCI) and Watts Factor (WF) (7).

Schafer nomogram has been used to differentiate patients with DU from patients with BOO (7, 8). BCI is calculated using a simple formula and can be used as screening method for DU, although it may be a method that is not able to differentiate patients with DU from those with BOO (7, 9). Finally, WF is calculated by a mathematical formula that evaluates the power applied on the bladder per area unit, and calculates detrusor strength during an isovolumetric contraction (10, 11).

Considering the limitations of all mentioned methods, two methods which would have few interfering elements were selected: Bladder Contractility Index (BCI) and Watts Factor (WF) (4, 12).

Despite the fact that some evidence suggests symptoms and post void residual can indicate DU, the information is not well consolidated and the differentiation between DU and BOO remains a problem.

The aim of this study is to evaluate the validity of the lower urinary tract symptoms (LUTS), International Prostate Symptom Score (IPSS), BCI, WF and post void residual to differentiate DU from BOO.

## PATIENTS AND METHODS

Comparative retrospective cross-sectional observational study performed between two groups: Group 1, patients without BOO and with diagnosis of DU, Group 2, patients with BOO. Two phases were performed: the first one, in order to calculate the sample size, and the second, with the final results. Data from the study were obtained from the database of urodynamic exams performed and stored at the Urodynamic Service of HC-UNICAMP (Hospital das Clínicas of the University of Campinas), from patient's medical charts and from HC Computer System. This study was approved by the Ethics Committee in Research, number 1.644.754.

All patients were male and underwent urodynamics between 2011 and 2018. Criteria for inclusion in Group 1 were: absence of obstruction or doubt in ICS Nomogram and presence of weak detrusor in Schafer Nomogram. Inclusion criteria in Group 2 was presence of obstruction in ICS Nomogram. Exclusion criteria were: presence of neurological disease, postoperative of pelvic exenteration, bladder cancer, prostate cancer and patients who underwent prostatic surgery.

In the first phase of the study, 5% significance level (alpha or type I error), and 80% sample power (beta or 20% type II error) were used for sample size calculation. The calculation was made considering the comparison of variables between the two groups. For BCI and WF, 22 and 20 patients were respectively required in each group. Therefore, the sample was enlarged in order to obtain 22 patients in each group.

Besides age and comorbidities, symptoms were analyzed through the International Prostate Symptom Score (IPSS) and classified as storage, voiding, combination of storage and voiding symptoms, and urinary incontinence, urodynamic data: post void residual (PVR) and classification of ICS and Schafer nomograms and calculation of Bladder Contractility Index and Watts Factor.

Categorical variables were described using absolute frequency and percentage, and numerical variables were described as mean, median, standard deviation, minimum and maximum. Mann--Whitney test was used for numerical variables, Chi-square test for PVR and Fisher's exact test for symptoms, considering a 5% significance level.

## RESULTS

A total of 44 patients were included in the study, 22 in each group. The average age was 68 years and 67.5 years for Group 1 and Group 2, respectively. (p=0.8416).

Arterial hypertension was present in the same proportion between groups (64%) (p=1.0), while Diabetes Mellitus appeared in 23% of patients in Group 1 and 36% in Group 2 (p=0.51). Dyslipidemia was present in 27% in Group 1 and 18% in Group 2 (p=0.72), and Acute Myocardial Infarction appeared in 23% and 5% patients in Groups 1 and 2, respectively (p=0.18), patients without comorbidities represented 17% in Group 1 and 18% in Group 2 (p=1.0, Fisher's exact test).

For IPSS, the following means were obtained: 16.5 (SD=5.76) for Group 1 and 20.5 (SD=6.94) for Group 2, without significant difference (p=0.858, Mann-Whitney test). For symptoms, the most prevalent category in both groups was the combination of storage and voiding symptoms with 12 patients in Group 1 and 15 patients in Group 2. However, there was not significant difference between groups (p=0.1810, Fisher's exact test).

Post void residual ≥30mL was found in 15 patients in Group 1 and 16 patients in Group 2, without significant difference (p=0.7411, Chi-Square test).

BCI presented median of 75 (SD=120.43) in Group 1, while in Group 2 median was 755.50 (SD=408.12), with significant difference (p <0, 0001, Mann-Whitney Test). For WF, Group 1 showed median of 22.42 (SD=14.88) and Group 2 median of 73.85 (SD=39.86), also with significant difference (p <0.0001, Mann-Whitney test). On the other hand, age and post void residual did not show statistically difference (p=0.8416 and p=0.5327, respectively) (Tables 1 and 2).

**Table 1:** Frequency of urodynamic data: measure of detrusor strength by Schafer Nomogram; values of BCI and WF, considering cut-off point values for Detrusor Underactivity.

VARIABLES			GROUP 1 - n (%)	GROUP 2 - n (%)
**Schafer**				
	Very weak		3 (13.64%)	1 (4.55%)
	Weak		18 (81.82%)	4 (18.18%)
Normal (−)			1 (4.55%)	–
Normal (+)			–	12 (54.55%)
Strong			–	5 (22.73%)
**BCI**				
	≤ 100		17 (77.27%)	2 (9.09%)
		> 100	5 (22.73%)	20 (90.91%)
**WF**				
	≤ 7		7 (31.82%)	2 (9.09%)
	> 7		15 (68.18%)	20 (90.91%)

**Table 2:** Descriptive and comparative analysis between groups: age, urodynamic data and post void residual; mean, median, standard deviation, minimum, maximum, and confidence intervals were described. There was a significant difference between groups for BCI and WF (p <0.0001; Mann-Whitney test).

VARIABLES	Age (years)		BCI		WF		PVR (mL)	
	Group 1	Group 2	Group 1	Group 2	Group 1	Group 2	Group 1	Group 2
**N**	22	22	22	22	22	22	22	22
**Mean**	68.23	67.5	125.41	782.82	21.3	73.33	99.36	134.77
**Median**	68	67.5	75	755.5	22.42	73,85	85	125
**Standard Deviation**	10.85	9.29	120.43	408.12	14.88	39.86	93.43	146.18

**Minimum**	50	45	43	68	1.66	2.73	0	0
**Maximum**	86	83	528	1677	51.37	182	350	620
**p**	0.8416		<0.0001		<0.0001		0.5327	

**BCI** = Bladder Contractility Index; **WF** = Watts Factor; **PVR** = Post Void Residual

## DISCUSSION

In a recent study comparing patients with DU and BOO, age was not different between groups, as in our study (13). However, it is worth noting that literature shows that DU is more common in the elderly, as demonstrated here, with median age of 68 years (4, 13-15).

Regarding comorbidities, a study comparing these two diseases also showed no difference between groups for hypertension, DM and other diseases, as demonstrated in this study (16). However, it is interesting to note that duration of hypertension (more than 10 years) and DM (more than 6 years) resulted in detrusor contractility changes (13). It is relevant because DM is a disease that leads to nerve damage and deposition of collagen and extracellular matrix in the detrusor, leading to changes on its function (17, 18).

IPSS score, classically described to evaluate the severity of prostate-related symptoms, is presented as a numerical index able to classify symptoms into categories such as mild, moderate or severe. As we have seen, Group 1 did not present statistically difference from Group 2, such as demonstrated by other authors who compared groups of patients with DU and BOO using IPSS score (14). Meanwhile, another study showed a correlation between DU and IPSS, with an association of IPSS between 20-23 and DU (13). Therefore, there is not consensus on validity of IPSS score and its cut-off to DU.

Regarding symptoms, their combination was predominant in both groups in this study, however, an interesting fact is that no patient had only storage symptoms in Group 2, but this information was not significant. Corroborating this information, another study comparing men with DU and BOO showed no difference in symptoms between groups (19). Thus, it seems that the category of symptoms alone cannot differentiate these diagnoses, but it needs to be investigated in further studies.

For BCI, literature places the cut-off value as 100. According to these criteria, BCI <100 corresponds to weak detrusor, between 100-150, normal detrusor, and >150, strong detrusor (12). As observed in Table 2, there is a statistically difference between the groups for BCI, showing that this could be a good parameter, in opposition to what has been presented in other articles that BCI is not able to differentiate patients with DU from those with BOO (8, 20).

The second parameter, Watts Factor, also presented statistical difference between the groups, so it could be a good indicator as well. For WF, literature suggests the value of 7W/m2 as a limit of normality (10). However, although this cut-off has been suggested to the diagnosis of DU, it could be a parameter to differentiate patients with DU from those with BOO (20).

Taking into account the results of our study we suggest the cut-offs of BCI and WF to classify DU and BOO could vary from the classical cut-offs of 7W/m2 to WF and 100 to BCI. However, at present, this cannot be accepted as truth, but should be assessed in future studies.

One study compared Schafer Nomogram, BCI and WF, and showed that only Schafer Nomogram would be able to identify BOO, however, we could observe from the data presented that both BCI and WF were able to differentiate DU from BOO (8).

In another study, there was a correlation among BCI, WF and BOO, inferring that as the degree of bladder outlet obstruction increases, BCI and WF values would increase proportionally. In this line, the authors of that study question the cut-offs used in the diagnosis of DU, suggesting that low values (BCI <100 and WF <7) would be present since there is not BOO and the detrusor muscle is not required to increase its strength in order to compensate resistance. However, when there is DU, there is no increase in detrusor pressure, since there is detrusor failure. Nevertheless, as the study itself presented, there was statistical difference for BCI and WF when compared to Bladder Outlet Obstruction. They questioned both the DU diagnosis criteria and the possible parameters that would compare DU with BOO, but affirmed that these were initial conclusions and needed to be validated (14). One factor to be taken into account is that this compensatory increase could reflect the initial phase of the disease, with the detrusor muscle is still strong, and the second moment, in the evolution of the disease, when it would lose its strength (21). However, in the current studies, there is not reference on the possible DU evolution time and this should be considered in future studies.

One study demonstrated that patients with DU presented high PVR when compared to the control group. In this study, for the diagnosis of DU, the cut-off was 147mL, with sensitivity and specificity of 60.16% and 72.97%, respectively, furthermore, these authors showed that PVR was an independent predictor for DU (13). Although more data suggest that residual volume >40% maximum cystometric capacity would be a strong indicator of DU, it is not a consensus (4). Here, PVR did not show statistical difference between groups, indicating that PVR alone would not be able to differentiate diagnoses, as demonstrated in other studies, which also did not show significant difference between Obstructed and Non-obstructed (14, 19). Thus, although PVR can be calculated using a non-invasive method, such as abdominal ultrasonography, it cannot yet be considered a reliable diagnosis factor, requiring further studies.

The limitations of this study were the small number of patients, the quality of data collected, some missing data, because it is a retrospective study, and the need to classify the symptoms into categories, rather than considering each one separately, because the data collected was registered in categories of symptoms.

## CONCLUSIONS

It is possible to conclude that the lower urinary tract symptoms, the International Prostate Symptom Score and post void residual did not show difference between Detrusor Underactivity and Bladder Outlet Obstruction in this study, in contrast, Watts Factor and Bladder Contractility Index were relevant tools in this differentiation.
